# Estimation of normal lung weight index in healthy female domestic pigs

**DOI:** 10.1186/s40635-023-00591-7

**Published:** 2024-01-26

**Authors:** Antonio Fioccola, Rosmery Valentina Nicolardi, Tommaso Pozzi, Isabella Fratti, Federica Romitti, Francesca Collino, Verena Reupke, Gianluigi Li Bassi, Alessandro Protti, Alessandro Santini, Massimo Cressoni, Mattia Busana, Onnen Moerer, Luigi Camporota, Luciano Gattinoni

**Affiliations:** 1 IRCCS San Raffaele Scientific Institute, Milan, Italy; 2 Department of Health Sciences, University of Florence, Florence, Italy; 3 Department of Anesthesiology, University Medical Center Göttingen, Göttingen, Germany; 4 Department of Health Sciences, University of Milan, Milan, Italy; 5 Department of Surgical Sciences, University of Turin, Turin, Italy; 6 Department of Experimental Animal Medicine, University of Göttingen, Göttingen, Germany; 7 Critical Care Research Group, The Prince Charles Hospital, Brisbane, QLD Australia; 8 Prince Charles Hospital Northside Clinical Unit, Faculty of Medicine, University of Queensland, Brisbane, QLD Australia; 9 Uniting Care Hospitals, Intensive Care Units St Andrew’s War Memorial Hospital and The Wesley Hospital, Brisbane, QLD Australia; 10 Wesley Medical Research, Brisbane, QLD Australia; 11 Queensland University of Technology, Brisbane, QLD Australia; 12 Department of Biomedical Sciences, Humanitas University, Pieve Emanuele, Milan Italy; 13 Department of Anesthesia and Intensive Care Units, IRCCS Humanitas Research Hospital, Rozzano, Milan Italy; 14 Unit of Radiology, IRCCS, Policlinico San Donato, San Donato Milanese, Milan Italy; 15 Department of Adult Critical Care Guy’s & St Thomas’ NHS Foundation Trust, London, UK; 16 Centre for Human & Applied Physiological Sciences, School of Basic & Medical Biosciences, King’s College London, London, UK

## Abstract

**Introduction:**

Lung weight is an important study endpoint to assess lung edema in porcine experiments on acute respiratory distress syndrome and ventilatory induced lung injury. Evidence on the relationship between lung–body weight relationship is lacking in the literature. The aim of this work is to provide a reference equation between normal lung and body weight in female domestic piglets.

**Materials and methods:**

177 healthy female domestic piglets from previous studies were included in the analysis. Lung weight was assessed either via a CT-scan before any experimental injury or with a scale after autopsy. The animals were randomly divided in a training (*n* = 141) and a validation population (*n* = 36). The relation between body weight and lung weight index (lung weight/body weight, g/kg) was described by an exponential function on the training population. The equation was tested on the validation population. A Bland–Altman analysis was performed to compare the lung weight index in the validation population and its theoretical value calculated with the reference equation.

**Results:**

A good fit was found between the validation population and the exponential equation extracted from the training population (RMSE = 0.060). The equation to determine lung weight index from body weight was: ${\text{Lung}} {\text{Weight}} {\text{Index}} \left(\frac{{\text{g}}}{{\text{kg}}}\right)=26.26*{10}^{-0.011*{\text{Body}} {\text{Weight}} \left({\text{kg}}\right)}.$ At the Bland and Altman analyses, the mean bias between the real and the expected lung weight index was − 0.26 g/kg (95% CI − 0.96–0.43), upper LOA 3.80 g/kg [95% CI 2.59–5.01], lower LOA − 4.33 g/kg [95% CI = − 5.54–(− 3.12)].

**Conclusions:**

This exponential function might be a valuable tool to assess lung edema in experiments involving 16–50 kg female domestic piglets. The error that can be made due to the 95% confidence intervals of the formula is smaller than the one made considering the lung to body weight as a linear relationship.

**Supplementary Information:**

The online version contains supplementary material available at 10.1186/s40635-023-00591-7.

## Introduction

Pigs are widely employed as experimental animals for the study of human pathologies such as sepsis or acute respiratory distress syndrome (ARDS), as well as to evaluate the effect of mechanical ventilation on the lung [1–6]. In these settings, the lung weight is an important marker of lung edema and therefore it is used to quantify the severity of lung injury. However, depending on the experimental model, the weight of the animal may range between 20 and 60 kg [1–4, 6–9]. These wide variations in lung weight make comparisons among different experimental results potentially inaccurate.

To allow more meaningful comparisons, lung weight is commonly normalized based on the actual body weight of the animal. Unfortunately, there are no data on the normal relationship between animal body weight and their lung weight, and physiological considerations [10] suggest that this relationship is unlikely to be linear, and therefore constant for the range of body weights. Indeed, an increase in age, muscles and body fat accumulation increases the body weight independently from the weight of the lung [11, 12].

To minimize this biological variability, experimental studies are usually designed to include animals with similar body weight. However, this is not always the case and, on several occasions, farms provide animals with body weight outside the requested range. This unanticipated variation can make the comparison of lung weights and severity of lung injury inaccurate.

In humans, there are different methods to estimate the normal lung weight, usually based on quantitative computed tomography (CT) scan analysis [13]. The CT has proved to be an excellent method of lung weight measurement also in pigs [14]. Therefore, both CT scan and direct measurement lung weight of healthy animals may be used to derive equations for the normalization of lung weight to body weight.

This approach could provide robust data on the physiological relationship between body weight and lung weight—which is currently lacking—and provide computational methods for the interpretation of experimental lung injury models.

Using data from several experiments involving healthy female domestic pigs, with body weight ranging from 16.5 to 52 kg (kg), [3, 15–22], the aim of this study is to determine the lung–body weight relationship in normal conditions and to provide a reference equation for normalization of lung weight in female domestic pigs with different body weight.

## Material and methods

### Study population

All the animals included in the current analysis are female piglets (White Landrace breed), from 12 previous experimental studies. The inclusion of pigs of female sex is due to the fact that it is the most commonly used animal [1–4, 6, 7, 23] mainly because of easier instrumentation and urethral catheterization. We only included healthy pigs, to investigate their lung/body weight relationship in physiological conditions.

Therefore, the lung weight was determined by CT scan, before any experimental injury, or by direct lung weight measurement using a scale, in pigs sacrificed immediately before the experiment or after non-injurious mechanical ventilation. Only animals confirmed to be healthy, i.e., with non-infected lungs after autopsy, were included. Details about inclusion process and reference studies/lung weighing method in each study are provided in Additional file 1: Figure S1 and in Table 1.

**Table 1:** Original studies from which the studied population was derived

Original study	Number of pigs	Timing of lung weight assessment	Lung weight method
Protti et al*.* [15]	29	Immediately after anesthesia induction	TC
Protti et al*.* [16]	28	Immediately after anesthesia induction	TC
Protti et al*.* [17]	5	Immediately after anesthesia induction	TC
Protti et al*.*[18]	30	Immediately after anesthesia induction	TC
Santini et al*.* [19]	10	Immediately after anesthesia induction	TC
Protti et al*.* [20]	31 (45 animals from [15, 16])	Immediately after anesthesia induction	TC
Romitti et al*.* [3]	8	Immediately after anesthesia induction	Scale
Li Bassi et al*.* [24]	12	After up to 72 h of protective mechanical ventilation	Scale
Amaro et al*.* [22]	6 (2 from unpublished data)	After up to 72 h of protective mechanical ventilation	Scale
Motos et al., abstract presented at ECCMID 2023, unpublished data	10	After up to 72 h of protective mechanical ventilation	Scale
Gattinoni et *al.,* unpublished data	3, originally assigned to Romitti et al*.* [3] and excluded from the analysis for technical problem during animals’ preparation	Immediately after anesthesia induction	Scale
Protti et al*.,* unpublished data	5, originally assigned to Protti et al*.* [15] and excluded from the analysis for technical problem during animals’ preparation	Immediately after anesthesia induction	Scale

### Lung weight assessment

Normal body weight was derived from CT scan or directly measured after autopsy, before experimental procedures (i.e., at baseline normal conditions), when pigs were not subjected to experimental injury or harmful mechanical ventilation.

#### CT-quantitative analysis

CT-scans were obtained at zero cmH_2_O of airway pressure with standardized settings (collimation, 5 mm; interval, 5 mm; bed speed, 15 mm/s; voltage, 140 kV; current, 240 mA; Lightspeed QXi, GE Healthcare, Madison, WI, USA). Quality controls were performed every month using standard phantoms. Each slide of the lung CT scan was manually contoured by experienced operators, to exclude proximal airways, large vessels and lymph-nodes, mediastinum, muscles, bones and pleural effusion. The image was therefore analysed with the Maluna software (Maluna 3.15, University Hospital of Göttingen, Germany). The lung weight was measured with the following formula:

$${\text{Tissue}}\;{\text{weight}} = \left[ {1 - \left( {\frac{{{\text{CT}}\;{\text{number}}}}{ - 1000}} \right)} \right] \times {\text{ Voxel}}\;{\text{volume}}$$

CT number was expressed in Hounsfield units (HU). Values of − 1000, 0 and + 1000 HU were assigned to air, lung tissue (including parenchyma, blood, and water) and bone, respectively. Voxel volume was 1.8 mm^3^. Lung tissue weight was the sum of the weight of all selected voxels [14, 25, 26].

Lung weight index was measured as

$${\text{Lung}}\;{\text{weight}}\;{\text{index}} \left( {\frac{{\text{g}}}{{{\text{kg}}}}} \right) = \frac{{{\text{lung}}\;{\text{weight}} \left( {\text{g}} \right)}}{{{\text{body}}\;{\text{weight}} \left( {{\text{kg}}} \right)}}$$

#### Autopsy

The autopsy was performed immediately after euthanasia and the lung weight was obtained using a scale. No exsanguination was intentionally performed before weighting the lungs.

### Statistical analysis

A regression analysis of body weight and the lung weight index (g/kg) was performed, to determine the best fitting model. We divided the populations in two groups: one used for derivation of the model (80% of the overall population) and the second to validate the model and the prediction of the formula (20% of the overall population). To further validate the reference equation, we performed a cross validation analysis. To assess the difference between the real lung weight in pigs belonging to the validation set and the ideal lung weight calculated with the reference equation, we performed a Bland and Altman analysis.

All statistical analyses were performed with R 4.2.3 (R Foundation for Statistical Computing, Vienna, Austria).

## Results

**Fig. 1:**
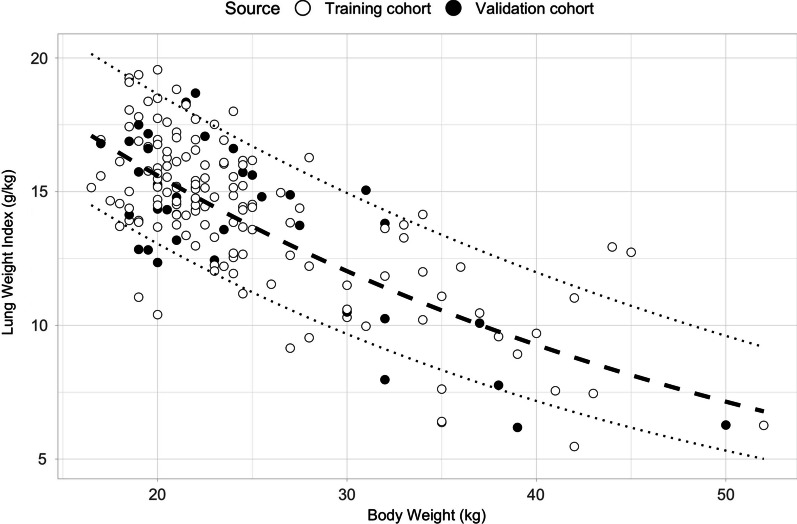
Body weight versus lung weight index regression and exponential function fitting the training population (white points) and piglets belonging to the validation population (black points). The exponential function is expressed by the black dashed line. 95% CI of the function are represented as black dotted lines. Upper 95% CI function: *Lung Weight Index* = 28.84 * 10 (− 0.009*Body Weight). Lower 95% CI function: *Lung Weight Index* = 23.44 * 10 (− 0.013*Body Weight). *g* grams, *kg* kilograms, *CI* confidence intervals

We studied 177 healthy pigs, retrospectively collected from 12 experimental studies. A complete flow-chart describing the process and population zoometric data are available in the supplementary material (Additional file 1: Figure S1, Table S2). In Fig. 1, we report the exponential function describing the lung weight index, expressed in g/kg, as a function of body weight (kg). The figure includes all animals, *i.e.,* pigs in the training cohort (*n* = 141, red dots), and pigs in the validation cohort (*n* = 36, green dots). The best fitting curve, following an exponential equation for the derivation population, was the following:

1 $${\text{Lung}}\;{\text{Weight}}\;{\text{Index}}\left( {{\text{g}}/{\text{kg}}} \right) = 26.26*10^{{ - 0.011*{\text{Body}}\;{\text{Weight}}\left( {{\text{kg}}} \right)}}$$

Exponential function explaining the relationship between lung weight index and body weight in a population of 141 female domestic piglets.

The correlation between the training population and the equation was good (RMSE: 0.068, MAE = 0.051).

When the equation obtained from the training population was tested against the validation population, we obtained a good correlation (RMSE = 0.070, MAE = 0.057) (Fig. 1). The results of the cross-validation analysis are similar to the results of the main analysis (see Additional file 1: Table S3 for details).

In Additional file 1: Figure S2, we thereby performed two additional population split (70%/30% and 90%/10%) on the same population, to further validate the results. In Additional file 1: Table S4, further details about the population composition (lungs weighed using the CT scan or the scale method) are available, together with the equations of the three different models (70/30, 80/20 and 90/10).

**Fig. 2:**
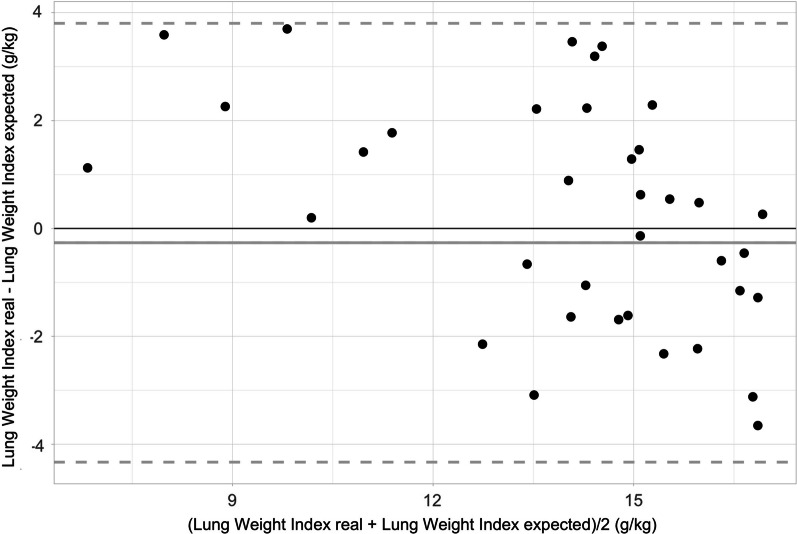
Bland and Altman comparing the real lung weight index of the validation population versus the expected lung weight index calculated with the formula. Mean bias was -0.26 g/kg (95% CI − 0.94–0.43), upper LOA = 3.80 (95%CI 2.72–4.82), lower LOA = − 4.33 (95% CI − 5.54–− 3.12). *g* grams, *kg* kilograms, *LOA* Level of agreement. Figure: black continuous line = mean bias; Black dashed lines = upper and lower LOA; red dotted lines = 95%CI of mean bias, upper and lower LOA

As additional test for the validity of Eq. 1, in Fig. 2 we show the Bland and Altman plot in which we compared the actual measured lung weight index in the validation population (*n* = 36) against the estimated lung weight, calculated using the Eq. 1 and the baseline body weight of the pigs. The points had an overall good distribution, with a mean bias of − 0.26 [95% CI − 0.96–0.43], an upper level of agreement (LOA) of 3.80 [95% CI 2.59–5.01] and a lower LOA of − 4.33 [95% CI − 5.54–(− 3.12)].

## Discussion

In this study, we aimed to investigate the relationship between lung and body weight in normal female pigs with weight ranging from 16.5 to 52 kg—a range frequently used in experimental studies of acute respiratory distress syndrome (ARDS), and ventilator-induced lung injury (VILI) (see Additional file 1: Table S1). We found that the lung–body weight relationship developed in the derivation population fits a negative exponential function and the resulting equation predicts the lung weight in the validation population with a high correlation. The 95% CI of the estimated parameters of the exponential regression proposed in the results’ section give us the possibility to discuss the possible advantages of using normalization of the normal expected lung weight instead of simply considering the actual body weight of the animal.

The normalization of the lung weight on the body weight after experimental procedures, as usually performed, has an intrinsic bias due to the non-linearity of the lung–body weight relationship. If we compare, as an example, two animals weighting 20 and 40 kg, with a normal lung weight of 316 and 381 g. If both animals undergo the same damage, the result would be a doubling of the lung weight to 632 and 762 g. If normalized to the body weight, the 20 kg animal would show, after the damage, a weight of 31.6 g/kg, versus 19.1 g/kg in the 40 kg animal. This would suggest that the damage in the 20 kg animal is 40% higher than the damage measured in the 40 kg animal (see Additional file 1: Table S2 for further details). In contrast, normalizing to the healthy lung weight, the measured damage will be equal in both animals (i.e., double lung weight index compared to the baseline normal conditions calculated using the Eq. 1).

This represents an ideal example, as the expected lung weight has quite large variations based on the equation errors. Indeed, taking into account the 95% CI of the estimates, the highest possible error in the 20 and 40 kg animals would, respectively, lead to a maximum underestimation of 16% and 22%, while the maximum possible overestimation would be 19.5% and 29% (see Additional file 1: Figure S3 and Table S6 for more details). Therefore, even in the worst scenario, the normalization through the estimated normal lung weight would lead to an error lower (29%) than the one normally obtained using a normalization through body weight (40%) (see Additional file 1: Tables S5, S6 and Figure S3).

In addition to the variability, a second limitation of the proposed equation is that it is strictly limited to weight between 16 and 45/50 kg, a value in which the equation approaches a plateau. Applying to higher values of body weight, the equation leads to a possible lung weight underestimation. Furthermore, the equation is limited to the female gender and to female White Landrace breed. It must be underlined, however, that the same sex choice is done by almost all the groups using pigs for experiments in ARDS (See Additional file 1: Table S1).

In this study, we combined lung weights of animals measured by CT-scan and lung weight directly measured with scale. It has been already shown in female domestic pigs that measuring the same lung with scale or CT-scan leads to an almost identical lung weight [14]. A possible discrepancy, however, occurs when the lung weight is measured in vivo by CT-scan compared to a lung weight measured after the autopsy due to possible blood losses. It must be noted, however, that large blood vessels are excluded by manual segmentation performed by expert investigators or semi-automatic/automatic methods [27, 28], and the amount of capillary blood included in the small arteries and capillaries should be in the order of few milliliters (mL). Indeed, in a normal adult man with a lung weight between 750 and 900 g, the capillary blood content is about 80 mL, i.e., 8–10% of the total lung weight [29]. In the worst-case scenario, the difference between lung weight measured in vivo by the CT-scan versus the lung weight measured at the autopsy should be in a similar range.

The animal population is composed by healthy animals before any experimental injurious procedure. In most of the cases (134 animals) the CT-scan data was taken within short time (1–2 h) from the start of the experiments, with animals anesthetized and mechanically ventilated. We cannot however exclude that anesthesia may have induced lung alterations. These should be represented to mild atelectasis in the dependent lung regions [30, 31] which should not alter the lung weight. In our population, we also included 28 animals from studies by Li Bassi et al. [24] and Amaro et al. [22] which underwent longer time of ventilation. We did that as the lungs appeared perfectly healthy at the autopsy, without any infection or other visible lesions (see Additional file 1: Figures S4 and S5). They adequately fit the equation, following the behavior of the whole study population.

## Conclusion

The equation we provided, despite its limitations, may be useful to estimate the normal expected lung weight in healthy female domestic pigs, within the weight range of 16–50 kg.

### Supplementary Information

**Additional file 1:** **Table S1.** A non-exhaustive list of recent experimental studies to evaluate lung damage inflicted by mechanical ventilation on healthy or pathological lung of porcine models.** Figure S1.** Study flow-chart. 45 animals were excluded from reference 20 because already included in references 15 and 16 of the main text. 18 animals were excluded from Romitti et al. [3] because their autopsy was performed after 48 hours of intentional harmful mechanical ventilation. 10 animals were excluded from reference 22 because they received intrabronchial instillation of bacterial agents, as well as the 12 animals excluded from Li Bassi et al. [24].** Table S2.** Zoometric data of the 177 pigs included in the study, divided on the method used to weigh the lung in the original studies.** Table S3.** Output of validity of the reference equation on the training set, validation set and from a cross validation performed using a machine learning approach. Legends. RMSE, Root Mean Square Error; MAE, Mean Absolute Error; AIC, Akaike Information Criterion.** Figure S2.**. Three different configurations to perform the machine learning analysis. Body weight versus lung weight index regressions and exponential functions fitting the training population (red points) and piglets belonging to the validation population (green points). The exponential function is expressed by the black dashed line. 95% CI of the function are represented as black dotted lines. Animals in which lung weight was measured using a CT scan are represented as triangles. Animals in which a scale was used are represented as circles.** Panel A**. 70/30% split. Equation: Lung Weight Index = 26.18*10(− 0.011*bw).** Panel B**. 80/20% split. Equation: Lung Weight Index = 26.26*10(− 0.011*bw).** Panel C**. 90/10% split. Equation: Lung Weight Index = 26.42*10(− 0.011*bw). Legends: g, grams; kg, kilograms; CI, confidence intervals; bw, body weight (kg).** Table S4**. Animals proportion based on the method used to weigh the lung in the training and validation set in the three different configurations presented in figure Sx. All the equations with the respective statistical outputs are shown.** Table S5.** Possible systematic error when estimating the lung damage simply looking at the lung weight index in a lung accumulating 100% of its baseline weight due to oedema during the experimental phase. In a 20 kgs pig, the lung weight index of a lung doubling its baseline weight would be 31,6 g/kg, whilst in a 40 kgs pig the same damage would lead to a 19,1 g/kg lung weight index. By simply comparing the two indexes, we obtain an error of 40 % ((31.6 – 19.1)/31.6 = 0.4).** Figure S3.** Prediction of the maximum fractional error due to the 95% CI of the estimates of the exponential function. The red, continuous lines show the maximum possible underestimation and overestimation of the formula (equation 1), estimating lung weight index from body weight, in a range from 16 to 45 kgs of body weight. Legends: CI, Confidence Interval; kg, kilograms.** Table S6.** Simulation of maximal possible underestimation/overestimation due to the 95% CI of the logarithmic equation. In the real situation, the ratio between the lung weight with 100% of lung damage and the expected lung weight would be 2 (624/312 or 742/371 in an animal of 20 versus 40 kgs of body weight, respectively). In situation 1 (see figure S1), the damage would be underestimated of 16 % [((624/373) – 2)/ 2 = -0.16]. In situation 2, the damage would be overestimated of 19% [((624/261) – 2)/2 = 0.19]. In situation 3, the damage would be underestimated of 22% [((742/479) – 2)/2 = -0.22]. In situation 4, the damage would be overestimated of 29% [(742/287)-2/2 = 0.29]. These errors are smaller than the one done when simply considering the lung weight index in animals of different body weight (see table S2). The percentages obtained from these calculations are graphically shown in figure S2, in a range from 16 to 45 kgs. Legends: kg, kilograms; g, grams.** Figure S4**. Histological images (x40 and x200 magnification) of control animals from reference 22 and 28 (Amaro et al., Li Bassi et al.). Findings in these animals only included mild bronchiolitis and interlobular septal oedema. The following histology scoring was used to assess the samples: grades 0, healthy lung no pneumonia; grade 1, purulent mucous plugging; grade 2, bronchiolitis; grade 3, pneumonia; grade 4, confluent pneumonia; and grade 5, abscessed pneumonia. Of note, descriptive statistics confirmed a mean ± SD score of 1.5 ± 1.6, median 0.5 and IQR of 0-3 for all the animals included in the studies. The control animals included in the study, who did not receive any intrabronchial instillation, had a lower score when compared to the overall population.All the animals in the control population where ventilated for up to 72 hours with a tidal volume lower than 10 mL/kg, aiming to reach driving pressures lower than 25 cmH2O. Driving pressures attained in the overall population are shown in figure S5.** Figure S5**. Driving pressures used in the overall animal population of reference 24 (Li Bassi et al).

## Data Availability

All data are available in data sets.
