# Supplementary material for: Estimation of normal lung weight index in healthy female domestic pigs
Source: Intensive Care Med Exp. 2024 Jan 26;12:6. doi: 10.1186/s40635-023-00591-7 (PMC10811311; doi:10.1186/s40635-023-00591-7)
Supplement: Supplementary file 1 — Additional file 1: Table S1. A non-exhaustive list of recent experimental studies to evaluate lung damage inflicted by mechanical ventilation on healthy or pathological lung of porcine models. Figure S1. Study flow-chart. 45 animals were excluded from reference 20 because already included in references 15 and 16 of the main text. 18 animals were excluded from Romitti et al. [3] because their autopsy was performed after 48 hours of intentional harmful mechanical ventilation. 10 animals were excluded from reference 22 because they received intrabronchial instillation of bacterial agents, as well as the 12 animals excluded from Li Bassi et al. [24]. Table S2. Zoometric data of the 177 pigs included in the study, divided on the method used to weigh the lung in the original studies. Table S3. Output of validity of the reference equation on the training set, validation set and from a cross validation performed using a machine learning approach. Legends. RMSE, Root Mean Square Error; MAE, Mean Absolute Error; AIC, Akaike Information Criterion. Figure S2.. Three different configurations to perform the machine learning analysis. Body weight versus lung weight index regressions and exponential functions fitting the training population (red points) and piglets belonging to the validation population (green points). The exponential function is expressed by the black dashed line. 95% CI of the function are represented as black dotted lines. Animals in which lung weight was measured using a CT scan are represented as triangles. Animals in which a scale was used are represented as circles. Panel A. 70/30% split. Equation: Lung Weight Index = 26.18*10(− 0.011*bw). Panel B. 80/20% split. Equation: Lung Weight Index = 26.26*10(− 0.011*bw). Panel C. 90/10% split. Equation: Lung Weight Index = 26.42*10(− 0.011*bw). Legends: g, grams; kg, kilograms; CI, confidence intervals; bw, body weight (kg). Table S4. Animals proportion based on the method used to weigh the lung in the training and [file 40635_2023_591_MOESM1_ESM.docx]

**Estimation of normal lung weight index in healthy domestic pigs**

**Additional material**

Table S1…………………………………………………………………………………………….2

Figure S1……………………………………………………………………………………………3

Table S2…………………………………………………………………….……………………….3

Table S3……………………………..………………………………………………………………4

Figure S2…………………………………………………………………………………………….4

Table S4……………………………………………………………………………………………..5

Figure S3……………………………………………………………………………………………6

Table S5……………………………………………………………………………………………..6

Figure S4……………………………………………………………………………………………7

Figure S5……………………………………………………………………………………………8

**Table S1**

| Reference | Aim | Induction of lung damage | Lung damage evaluation method | Pigs characteristics (n, weight) |
| --- | --- | --- | --- | --- |
| Retamal J *et al.* [4] PMID: 29528946. | To investigate correlation between lung regional strain and regional inflammation | Double-hit model | CT scan and PET [^18^F] fluoro-2-deoxy-D-glucose | 12 female domestic pigs  (5 controls) |
|  |  |  |  | 25.4 ± 3.5 Kg |
| Yoshida T *et al.* [7]. PMID: 29708892. | To investigate the effect of continuous negative abdominal pressure added to PEEP versus PEEP alone on dorsal atelectasis recruitment | Double-hit model | Wet to dry,  inflammation molecular markers | 10 female Yorkshire |
|  |  |  |  | 20.2 to 24.6 Kg |
| Scharffenberg M *et al.* [8] PMID: 34880770; | To investigate the association between MP and pulmonary inflammation | Lung lavage in prone and supine position (37°C; 35 ml/kg; lavage pressure approximately 30 cmH_2_O) | PET [^18^F] fluoro-2-deoxy-D-glucose | 8 female domestic pigs |
|  |  |  |  | 47.7 ± 8 Kg |
| Wittenstein J *et al.* [3] PMID: 32033744; | To investigate the relation between different ventilation with variable V_T_ and the pulmonary compared with standard protective, nonvariable ventilation. | Double- hit model | Wet to dry ratio  PET  Inflammation, fibrosis and endothelial damage cells molecular markes  PCR | 14 female german landrace domestic pigs |
|  |  |  |  | 35.26 ± 3.73 Kg |
| Katira BH *et al.* [9] | To compare Ppl gradient, ventilation distribution, and regional compliance between dependent and nondependent lungs, and to investigate the effect of PEEP during supination and pronation | Double- hit model | Physiological measures and CT-scans | 14 female |
|  |  |  |  | 36.2 ± 2.0 Kg |
| Romitti F *et al.* [20] | To find an absolute mechanical power to prevent ventilation lung injury | Mechanical ventilation | Wet-to-dry ratio  Lungs’ weight  Anatomo-patologhical evaluation | 26 female domestic pigs (8 control) |
|  |  |  |  | 29.5 ± 2.5 kg (40.6 ± 7.9 kg) |
| Collino F *et al.* [1] | To investigate the effects of increasing mechanical power by selectively modifying its positive end-expiratory pressure component. | Mechanical ventilation | Lungs’ wet-to-dry ratio,  lungs’ weight,  anatomo-patologhical evaluation | 36 female domestic pigs |
|  |  |  |  | 23.3 ± 2.3 kg |
| Vassalli F *et al.* [3] | To determine which component of the mechanical power correlates more with VILI | Mechanical ventilation | Lungs’ wet-to-dry ratio,  lungs’ weight,  anatomo-patologhical evaluation | 42 female domestic pigs |
|  |  |  |  | 24.2 ± 2.0 kg |

***Table S1*** *A non-exhaustive list of recent experimental studies to evaluate lung damage inflicted by mechanical ventilation on healthy or pathological lung of porcine models.*

**Figure S1**

*Figure S1. Study flow-chart. 45 animals were excluded from reference 20 because already included in references 15 and 16 of the main text. 18 animals were excluded from Romitti et al. [3] because their autopsy was performed after 48 hours of intentional harmful mechanical ventilation. 10 animals were excluded from reference 22 because they received intrabronchial instillation of bacterial agents, as well as the 12 animals excluded from Li Bassi et al. [24].*

**Table S2**

|  | TC method | Scale method |
| --- | --- | --- |
| N (% of the total) | 128 (72.3%) | 49 (27.7%) |
| Body weight (kg) | 22 ± 2.82 kg | 31.9 ± 8.87 kg |
| Lung weight (g) | 329 ± 45.1 g | 334 ± 86 g |
| Lung weight index (g/kg) | 15.3 ± 1.76 g/kg | 11 ± 3.18 kg |

*Table S2. Zoometric data of the 177 pigs included in the study, divided on the method used to weigh the lung in the original studies.*

|  | TC method | Scale method |
| --- | --- | --- |
| N (% of the total) | 128 (72.3%) | 49 (27.7%) |
| Body weight (kg) | 22 ± 2.82 kg | 31.9 ± 8.87 kg |
| Lung weight (g) | 329 ± 45.1 g | 334 ± 86 g |
| Lung weight index (g/kg) | 15.3 ± 1.76 g/kg | 11 ± 3.18 kg |

|  | TC method | Scale method |
| --- | --- | --- |
| N (% of the total) | 128 (72.3%) | 49 (27.7%) |
| Body weight (kg) | 22 ± 2.82 kg | 31.9 ± 8.87 kg |
| Lung weight (g) | 329 ± 45.1 g | 334 ± 86 g |
| Lung weight index (g/kg) | 15.3 ± 1.76 g/kg | 11 ± 3.18 kg |

**Table S3**

|  | RMSE | MAE | AIC |
| --- | --- | --- | --- |
| Training set | 0.068 | 0.051 | -350.75 |
| Validation set | 0.070 | 0.057 | -84.62 |
| Cross validation | 0.070 | 0.053 | - |

*Table S3. Output of validity of the reference equation on the training set, validation set and from a cross validation performed using a machine learning approach. Legends. RMSE: Root Mean Square Error; MAE: Mean Absolute Error; AIC: Akaike Information Criterion.*

**Figure S2**


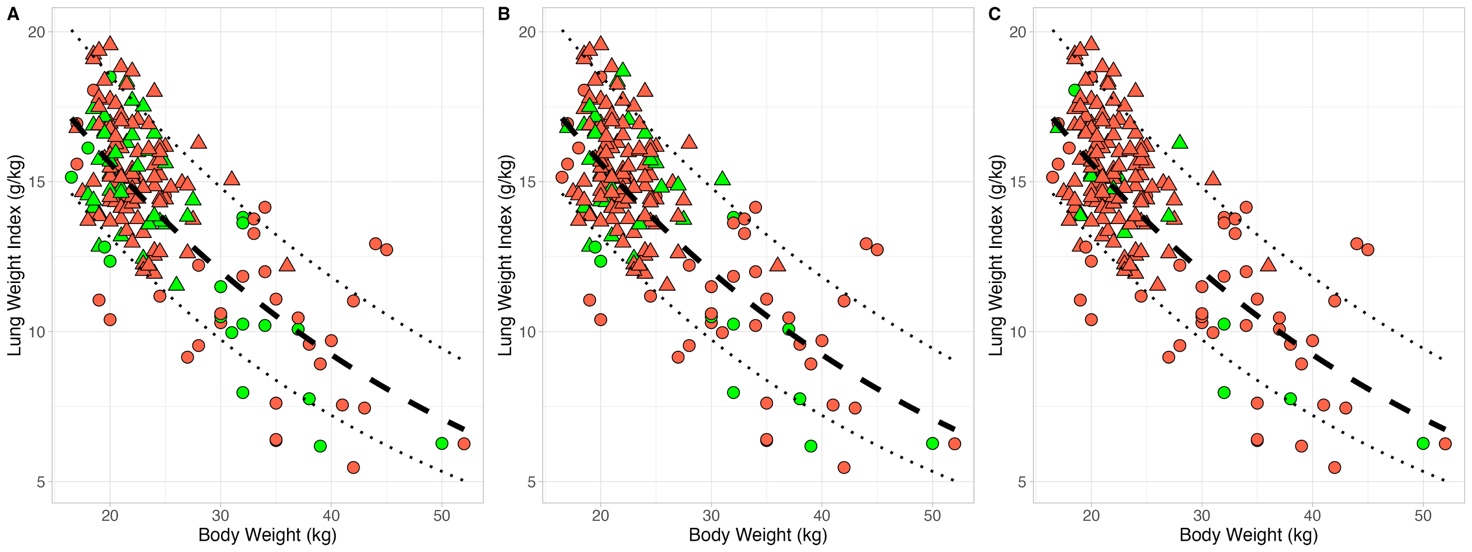


Figure S2. Three different configurations to perform the machine learning analysis. Body weight versus lung weight index regressions and exponential functions fitting the training population (red points) and piglets belonging to the validation population (green points). The exponential function is expressed by the black dashed line. 95% CI of the function are represented as black dotted lines. Animals in which lung weight was measured using a CT scan are represented as triangles. Animals in which a scale was used are represented as circles. Panel A. 70/30% split. Equation: Lung Weight Index = 26.18*10^(-0.011*bw)^. Panel B. 80/20% split. Equation: Lung Weight Index = 26.26*10^(-0.011*bw)^. Panel C. 90/10% split. Equation: Lung Weight Index = 26.42*10^(-0.011*bw)^. Legends: g = grams; kg = kilograms; CI = confidence intervals; bw = body weight (kg).

**Table S4**

| Split |  | 70/30 (panel A) | 80/20 (panel B) | 90/10 (panel D) |
| --- | --- | --- | --- | --- |
| Training set | **TC (N, %)** | 93 (**74.4%**) | 105 (**72.9%**) | 117 (**72.7 %**) |
|  | **Scale (N, %)** | 32 (**25.6 %**) | 39 (**27.1 %**) | 44 (**27.3%**) |
| Validation set | **TC (N, %)** | 35 (**67.3 %**) | 23 (**69.7 %**) | 11 (**68.8%**) |
|  | **Scale (N, %)** | 17 (**32.7 %**) | 10 (**30.3 %**) | 5 (**31.2%**) |
| Equation (lung weight index, g/kg) |  | 26.17*10^(-0.011*bw)^ | 26.26*10^(-0.011*bw)^ | 26.36*10^(-0.011*bw)^ |
| R^2^ (validation set) |  | 0.70 | 0.67 | 0.83 |
| RMSE (validation set) |  | 0.062 | 0.071 | 0.062 |
| MAE (validation set) |  | 0.049 | 0.058 | 0.046 |

*Table S4. Animals proportion based on the method used to weigh the lung in the training and validation set in the three different configurations presented in figure Sx. All the equations with the respective statistical outputs are shown.*

**Table S5**

| Body weight | Expected Lung Weight Index | Expected Lung Weight | Lung Weight after 100% damage | Lung Weight Index after 100% damage |
| --- | --- | --- | --- | --- |
| 20,00 | 15,82 | 316,46 | 632,93 | 31,65 |
| 30,00 | 12,28 | 368,48 | 736,96 | 24,57 |
| 40,00 | 9,53 | 381,38 | 762,75 | 19,07 |

*Table S5. Possible systematic error when estimating the lung damage simply looking at the lung weight index in a lung accumulating 100% of its baseline weight due to oedema during the experimental phase.
In a 20 kgs pig, the lung weight index of a lung doubling its baseline weight would be 31,6 g/kg, whilst in a 40 kgs pig the same damage would lead to a 19,1 g/kg lung weight index. By simply comparing the two indexes, we obtain an error of 40 % ((31.6 – 19.1)/31.6 = 0.4).*

**Figure S3**

**
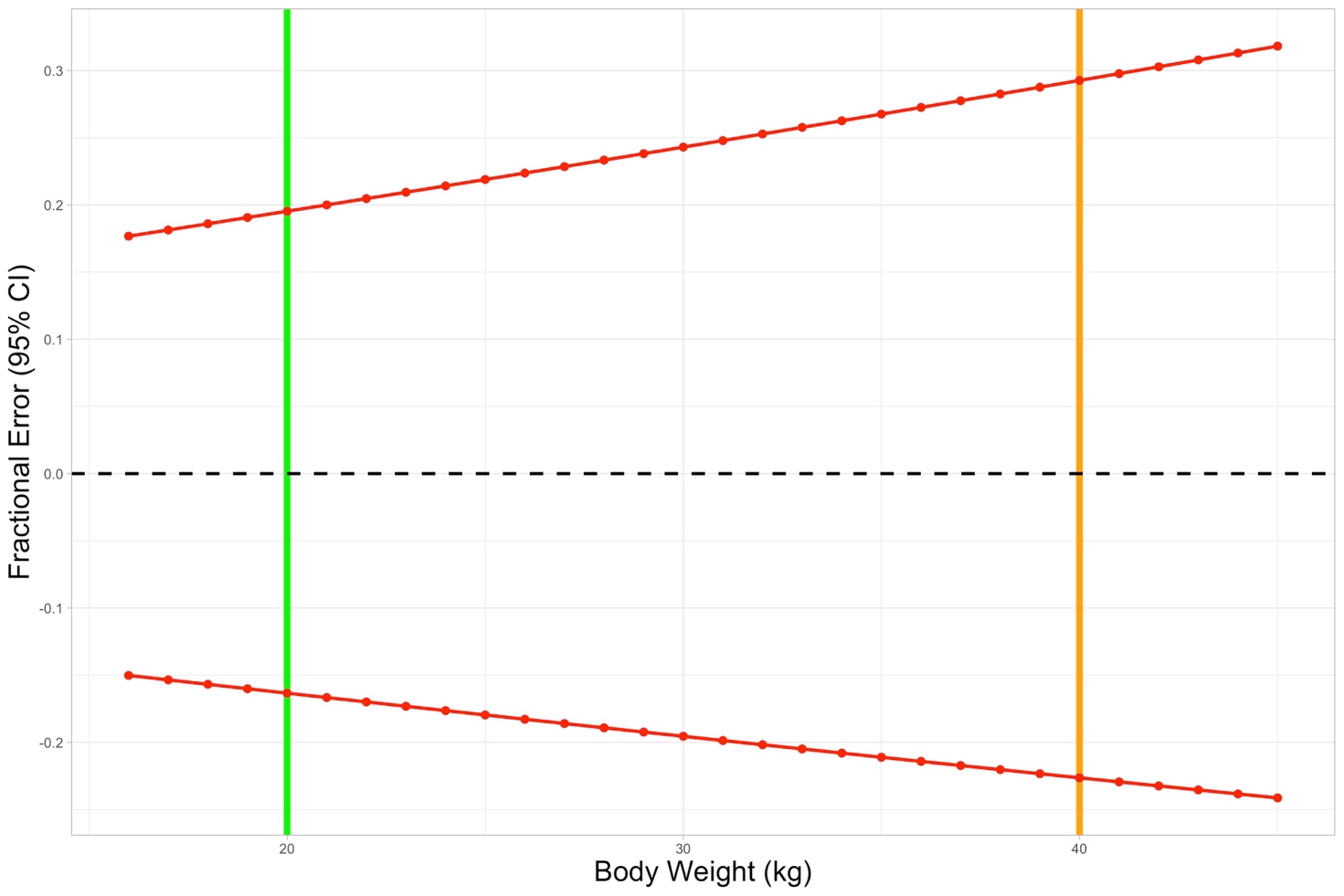
**

2

4

3

1

Figure S3. Prediction of the maximum fractional error due to the 95% CI of the estimates of the exponential function. The red, continuous lines show the maximum possible underestimation and overestimation of the formula (equation 1), estimating lung weight index from body weight, in a range from 16 to 45 kgs of body weight. Legends: CI = Confidence Interval; kg = kilograms.

**Table S6**

| Pig body weight (kg) | Expected lung weight (g) | Expected lung weight with 100% damage (g) | Lowest possible expected lung weight (95% CI) (g) | Highest possible expected lung weight (95% CI) (g) |
| --- | --- | --- | --- | --- |
| 20 | 312 | 624 | 261 | 373 |
| 40 | 371 | 742 | 287 | 479 |

Table S6. Simulation of maximal possible underestimation/overestimation due to the 95% CI of the logarithmic equation. In the real situation, the ratio between the lung weight with 100% of lung damage and the expected lung weight would be 2 (624/312 or 742/371 in an animal of 20 versus 40 kgs of body weight, respectively). In situation 1 (see figure S1), the damage would be underestimated of 16 % [((624/373) – 2)/ 2 = -0.16]. In situation 2, the damage would be overestimated of 19% [((624/261) – 2)/2 = 0.19]. In situation 3, the damage would be underestimated of 22% [((742/479) – 2)/2 = -0.22]. In situation 4, the damage would be overestimated of 29% [(742/287)-2/2 = 0.29]. These errors are smaller than the one done when simply considering the lung weight index in animals of different body weight (see table S2). The percentages obtained from these calculations are graphically shown in figure S2, in a range from 16 to 45 kgs. Legends: kg = kilograms; g = grams.

**Figure S4**

**
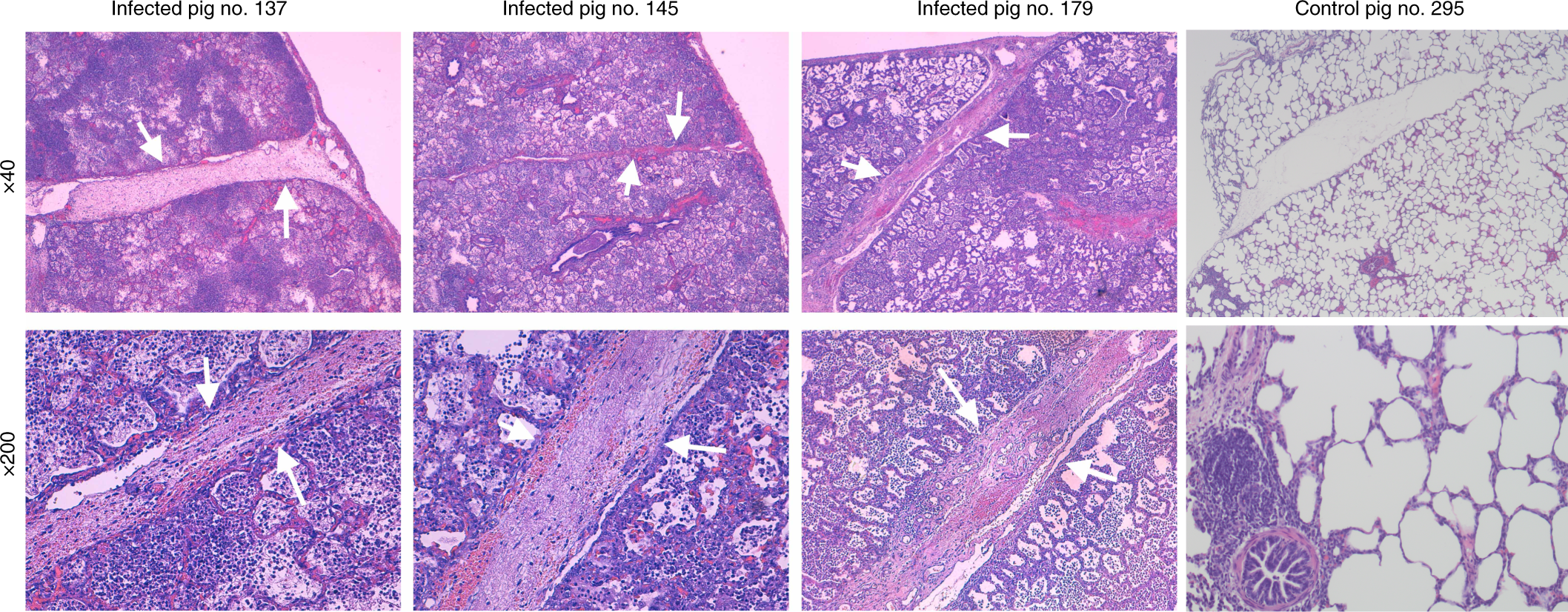
**

*Figure S4. Histological images (x40 and x200 magnification) of control animals from reference 22 and 28 (Amaro et al., Li Bassi et al.). Findings in these animals only included mild bronchiolitis and interlobular septal oedema. The following histology scoring was used to assess the samples: grades 0, healthy lung no pneumonia; grade 1, purulent mucous plugging; grade 2, bronchiolitis; grade 3, pneumonia; grade 4, confluent pneumonia; and grade 5, abscessed pneumonia. Of note, descriptive statistics confirmed a mean ± SD score of 1.5 ± 1.6, median 0.5 and IQR of 0-3 for all the animals included in the studies. The control animals included in the study, who did not receive any intrabronchial instillation, had a lower score when compared to the overall population.*

*All the animals in the control population where ventilated for up to 72 hours with a tidal volume lower than 10 mL/kg, aiming to reach driving pressures lower than 25 cmH_2_O. Driving pressures attained in the overall population are shown in figure S5.*

**Figure S5**

*Figure S5. Driving pressures used in the overall animal population of reference 24 (Li Bassi et al.).*
